# Advances in genetic factors of adolescent idiopathic scoliosis: a bibliometric analysis

**DOI:** 10.3389/fped.2023.1301137

**Published:** 2024-01-03

**Authors:** Xiaowei Jiang, Fuyun Liu, Mingxuan Zhang, Weiming Hu, Yufeng Zhao, Bing Xia, Ke Xu

**Affiliations:** Department of Orthopedics, The Third Affiliated Hospital of Zhengzhou University, Zhengzhou, Henan Province, China

**Keywords:** advances, genetic factors, adolescent idiopathic scoliosis, bibliometric analysis, CiteSpace

## Abstract

**Objective:**

This study offers a bibliometric analysis of the current situation, hotspots, and cutting-edge domains of genetic factors of adolescent idiopathic scoliosis (AIS).

**Methods:**

All publications related to genetic factors of AIS from January 1, 1992, to February 28, 2023, were searched from the Web of Science. CiteSpace software was employed for bibliometric analysis, collecting information about countries, institutions, authors, journals, and keywords of each article.

**Results:**

A cumulative number of 308 articles have been ascertained. Since 2006, publications relating to genetic factors of AIS have significantly increased. China leads in both productivity and influence in this area, with the Chinese Academy of Medical Sciences being the most productive institution. The most prolific scholars in this field are Y. Qiu and Z. Z. Zhu. The publications that contributed the most were from *Spine* and *European Spine Journal*. The most prominent keywords in the genetic factors of AIS were “fibrillin gene”, “menarche”, “calmodulin”, “estrogen receptor gene”, “linkage analysis”, “disc degeneration”, “bone mineral density”, “melatonin signaling dysfunction”, “collagen gene”, “mesenchymal stem cell”, “*LBX1*”, “promoter polymorphism”, “Bone formation”, “cerebrospinal fluid flow” and “extracellular matrix”.

**Conclusion:**

This analysis provides the frontiers and trends of genetic factors in AIS, including relevant research, partners, institutions and countries.

## Introduction

Adolescent idiopathic scoliosis (AIS) is a tridimensional structural abnormality of the spine; that impacts adolescents from 10 years old to maturity (1, 2). The main diagnosis is based on the coronal curved Cobb angle, which must be >10° on standard anterior and posterior radiographs (3). AIS affects 1%–4% of teenagers (2), and is more common in girls (3). It can lead to high and low shoulders, trunk displacement, and other appearance deformities (2, 4). The development of AIS involves a variety of factors such as genetics, tissues, spinal biomechanics, and hormones (5). Of these, genetics is the major factor. Early identification of relevant genetic variants can help in the diagnosis and prevention of the disease.

Bibliometrics evaluates published research and predicts research trends (6). It can analyze scientific movements, including relationships among countries, institutions, authors, journals, and keywords (7), and has been widely used in various fields. The knowledge map examines the progress and boundaries of the discipline, facilitating researchers to understand the research hotspots and guiding them in their research directions.

Although various studies have focused on the genetic factors of AIS, bibliometric analysis remains scarce. The purpose of this study is to organize relevant studies of AIS genetic factors from January 1, 1992, to February 28, 2023. It summarizes genetic factors and provides researchers with a macro perspective in this area of research. The study includes analysis of the quantity of published articles, associations and symbioses between authors or countries, co-citation of references, and hot areas associated with keywords in the global research and analysis of genetic factors of AIS.

## Method

### Data sources

All data are obtained from the core collection of the Internet database Web of Science (WoS). The literature on genetic factors of AIS published from January 1, 1992, to February 28, 2023, was searched on WoS. Retrieval strategy: TS = (“adolescent idiopathic scoliosis”) and TS = (gene or DNA or “base sequence” or “nucleic acid” or “copy number variants” or “single nucleotide polymorphism” or “SNP”). The database is updated daily and data are collected in a 24-hour period to prevent potential bias. Primary researches and literature reviews were included. Letters, corrections, meeting abstracts, editorial materials, proceeding papers and book chapters were excluded.

### Data analysis

Two separate evaluators (X.W.J. and M.X.Z.) conducted a comprehensive review of the study based on the title and abstract. The compilation and export of references and citations were done in a simple text format. Every bibliographic entry includes a title, author, keywords, abstract and references.

CiteSpace (a Java-based software) was used to perform a bibliometric analysis of the WoS Core Collection. It visualizes information and is frequently employed for evaluating patterns in research (7). It is capable of recognizing leading authors, institutions, and nations, and forming collaborative research connections among these entities. To examine research collaborative connections, a network analysis of references, scholars, and articles using co-citation was proceeded. Extend the co-word network analysis of keywords to provide up-to-the-minute viewpoints and research trends. Co-citation relationships show the recurrence with which a terminology or citation is mentioned over a given period. Co-occurrence surges manifest the recurrence with which a keyword or citation appears over a period of time.

The dimensions of the nodes in the visual network graph represent the extent of co-occurrence or recurrence. The line between nodes is the relationship of cooperation, co-occurrence and co-citation. The width and the extent of the lines indicate the proximity of cooperation between nations, organizations, and scholars. The lines depict the relationship among the nodes. Their color shows the publication year.

This study is primarily descriptive. Absence of statistics analysis, the quantity and proportion (%) of every index demonstrates the dispersion and change trends of different years, countries, organizations, publications, and scholars.

## Results

### Publication outputs

**Figure 1:**
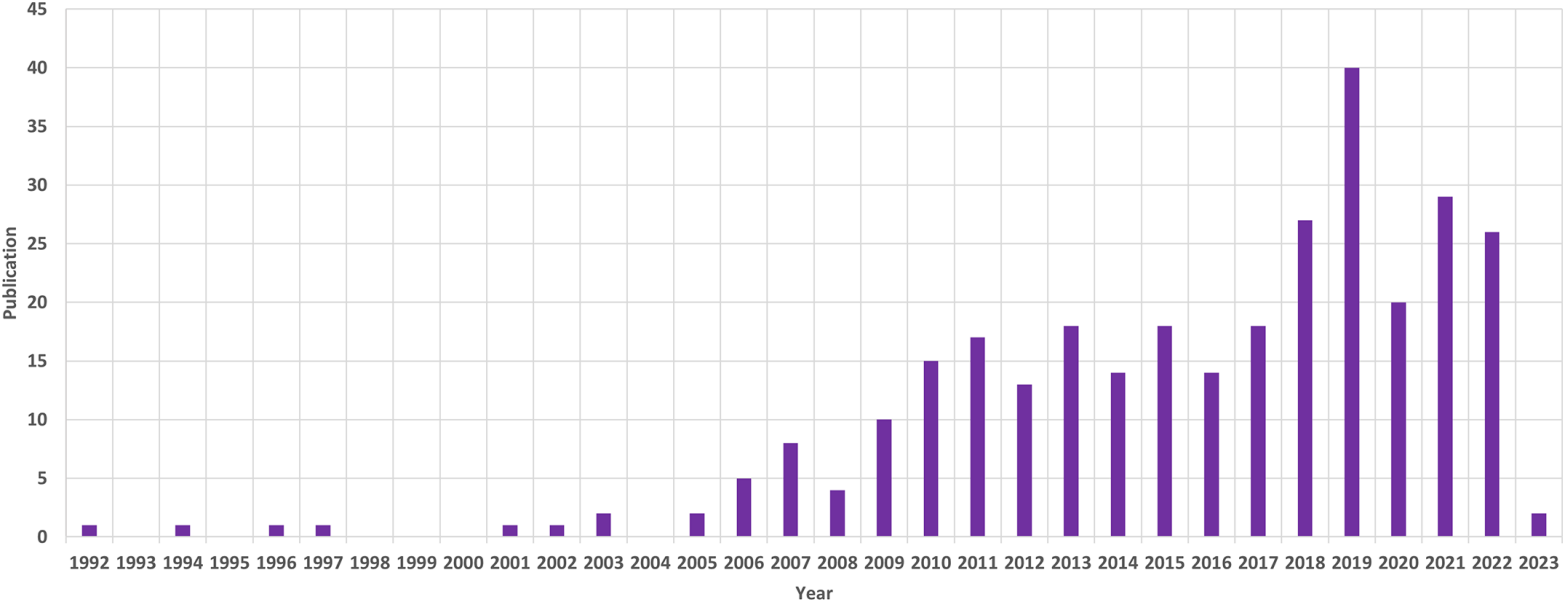
The annual patterns of publications.

Between January 1, 1992, and February 28, 2023, we retrieved a total of 330 records, of which 308 articles were eligible based on inclusion and exclusion criteria. With this group, there were 270 articles classified as original research and 38 articles categorized as reviews. English publications overwhelmingly dominated, accounting for more than 99% (306), while French and German publications were represented by only 1 paper each. From 1992 to 2022, research on AIS genetic factors exhibited a consistent upward trend, with an annual growth rate of 5.93%. The year 2019 stood out as the peak, with the highest number of papers at 40 (Figure 1). Initially, a limited number of publications were observed from 1992 to 2005, but since 2006, there has been a rapid increase. In total, 308 publications garnered 5,771 citations.

### Country/region distribution

**Figure 2:**
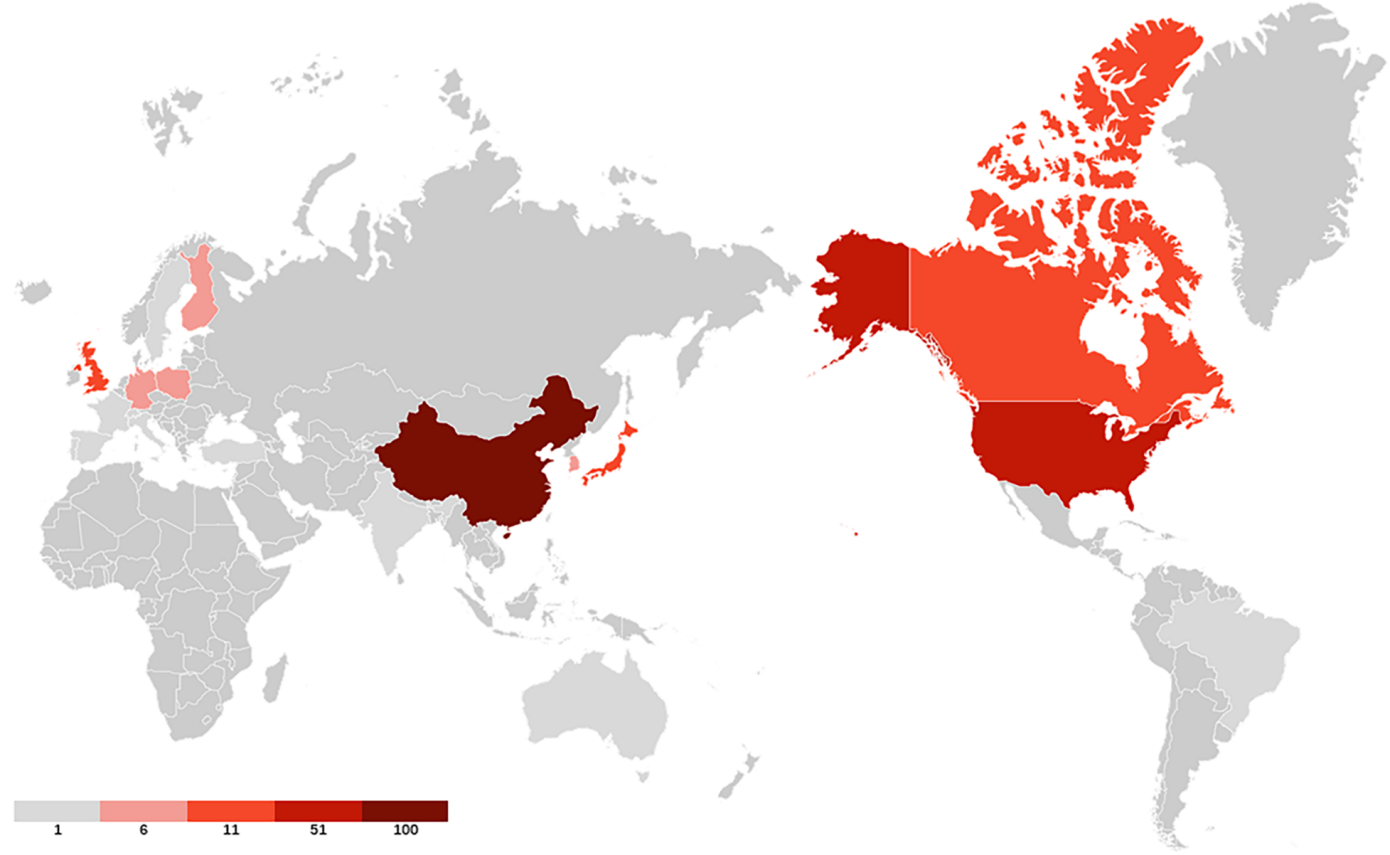
The geographic distribution of publications. Darker colors indicate greater number of publications.

**Figure 3:**
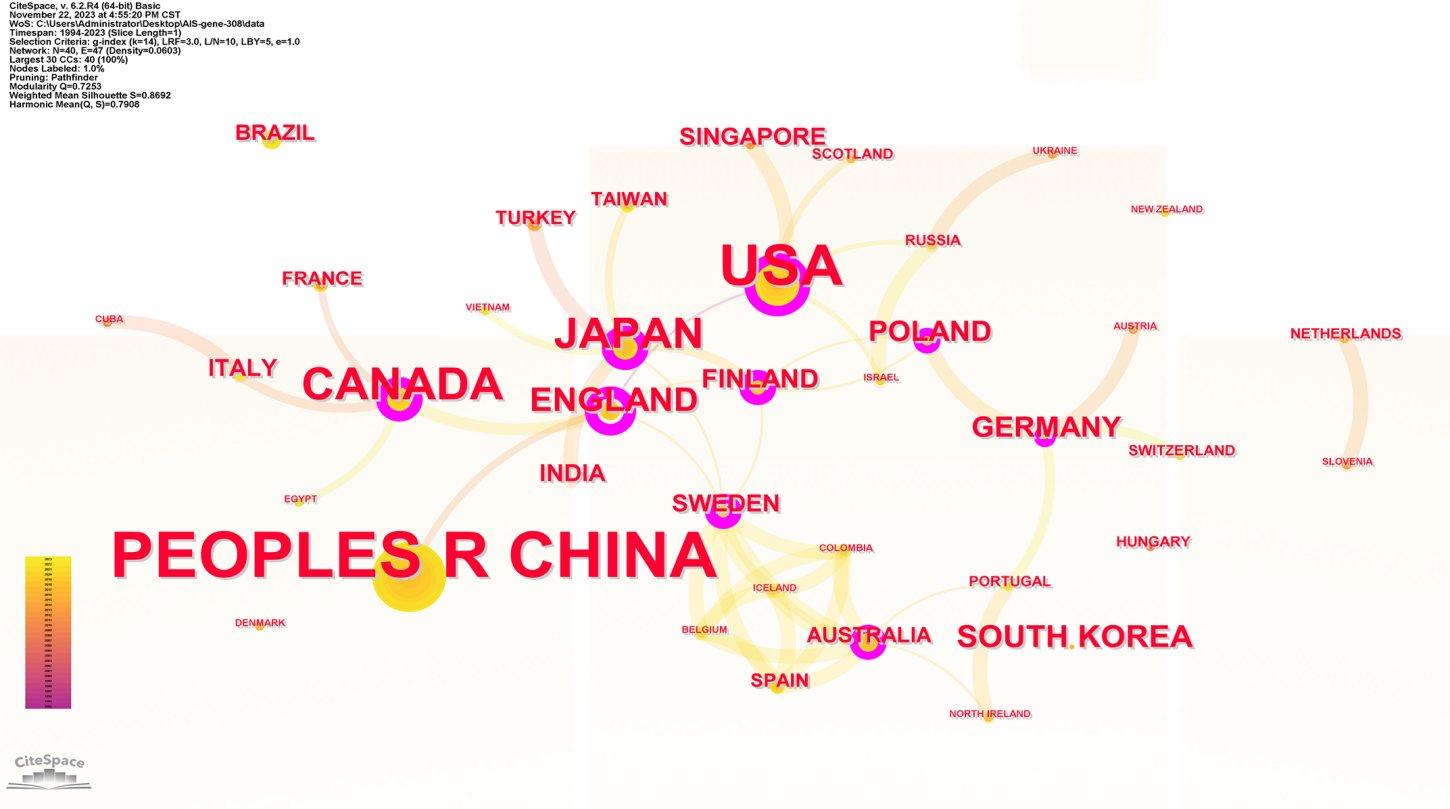
Collaboration network of productive countries/regions. Nodes represent countries/regions, and lines indicate co-citation relationships. Node colors correspond to different years. Larger nodes indicate higher publication numbers.

40 countries have published articles on AIS genetic factors. China was involved in the publication of most articles (50.3%), followed by the United States (25%), Canada (9.4%), Japan (8.8%) and the United Kingdom (4.2%). Figure 2 shows the geographic distribution of publications. Figure 3 shows the cooperation between different regions. Table 1 shows that the H-index (a common indicator of academic influence) (8) of China, the United States, Canada, Japan and the United Kingdom is 28, 27, 14, 18 and 7 respectively.

**Table 1:** Top 5 countries contributed to research publications on the genetic factors of AIS.

Rank	Country	Number	Percentage	H-index
1	China	155	50.3	28
2	United States	78	25.0	27
3	Canada	29	9.4	14
4	Japan	27	8.8	18
5	England	13	4.2	7

### Institution analysis

**Figure 4:**
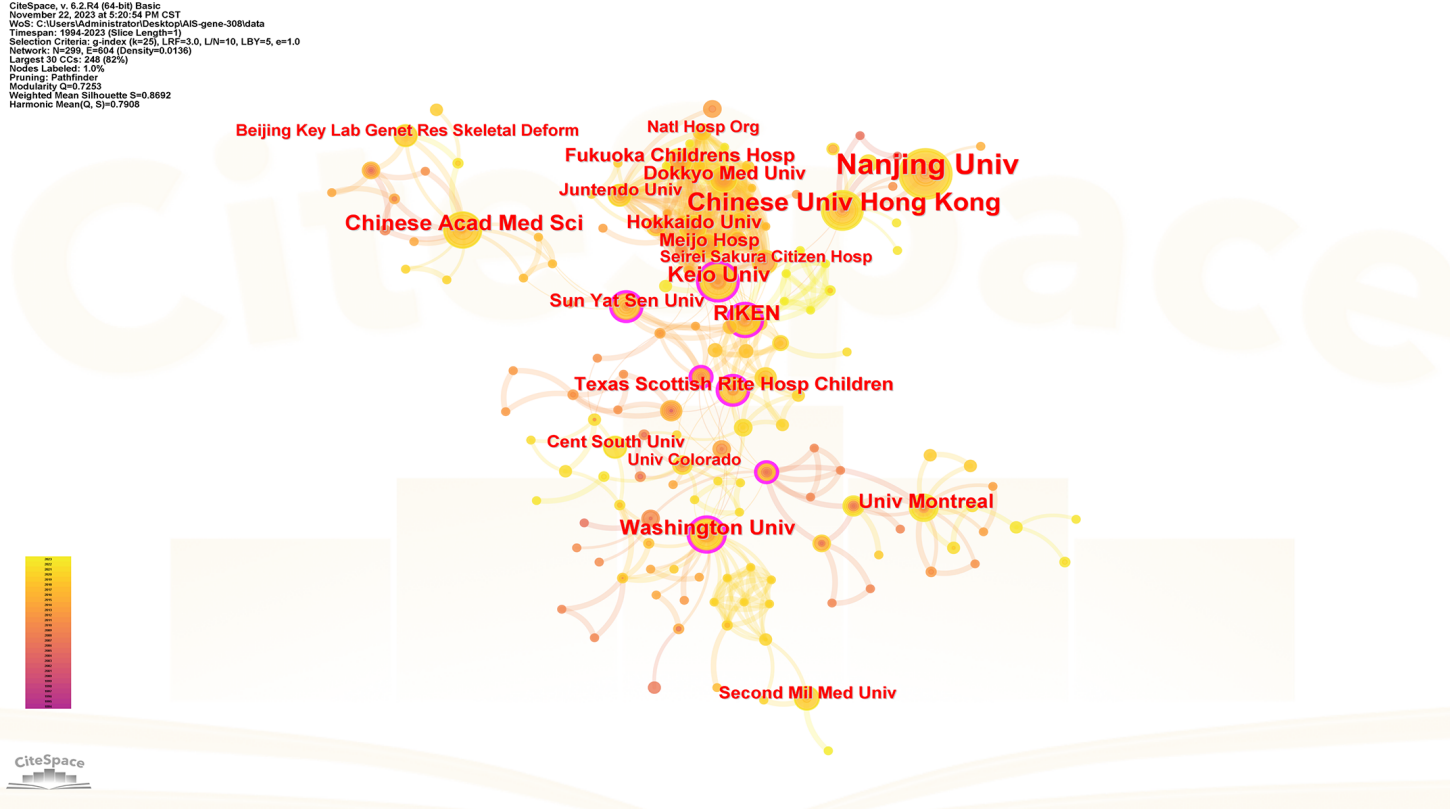
Collaboration network of productive institutions. Nodes represent institutions, lines represent co-citation relationships. Node colors indicate different years. Node size reflects publication numbers.

Table 2 shows the ranking of institutions involved in publishing articles on genetic factors in AIS. Chinese Academy of Medical Sciences was involved in the largest number of published papers (69; 22.4%), followed by Nanjing University (61; 19.8%), University of Hong Kong (30; 9.7%), RIKEN (21; 6.8%), University of Texas System (21; 6.8%). Figure 4 presents the cooperation among institutions in this field.

**Table 2:** Top 10 productive institutions in the genetic factors of AIS.

Rank	Institution	Number	Percentage
1	Chinese Academy of Medical Sciences	69	22.4
2	Nanjing University	61	19.8
3	University of Hong Kong	30	9.7
4	RIKEN	21	6.8
5	University of Texas System	21	6.8
6	Keio University	20	6.5
7	Washington University	20	6.5
8	University of Montreal	19	6.2
9	Central South University	17	5.5
10	Naval Medical University	15	4.9

### Journal analysis

**Figure 5:**
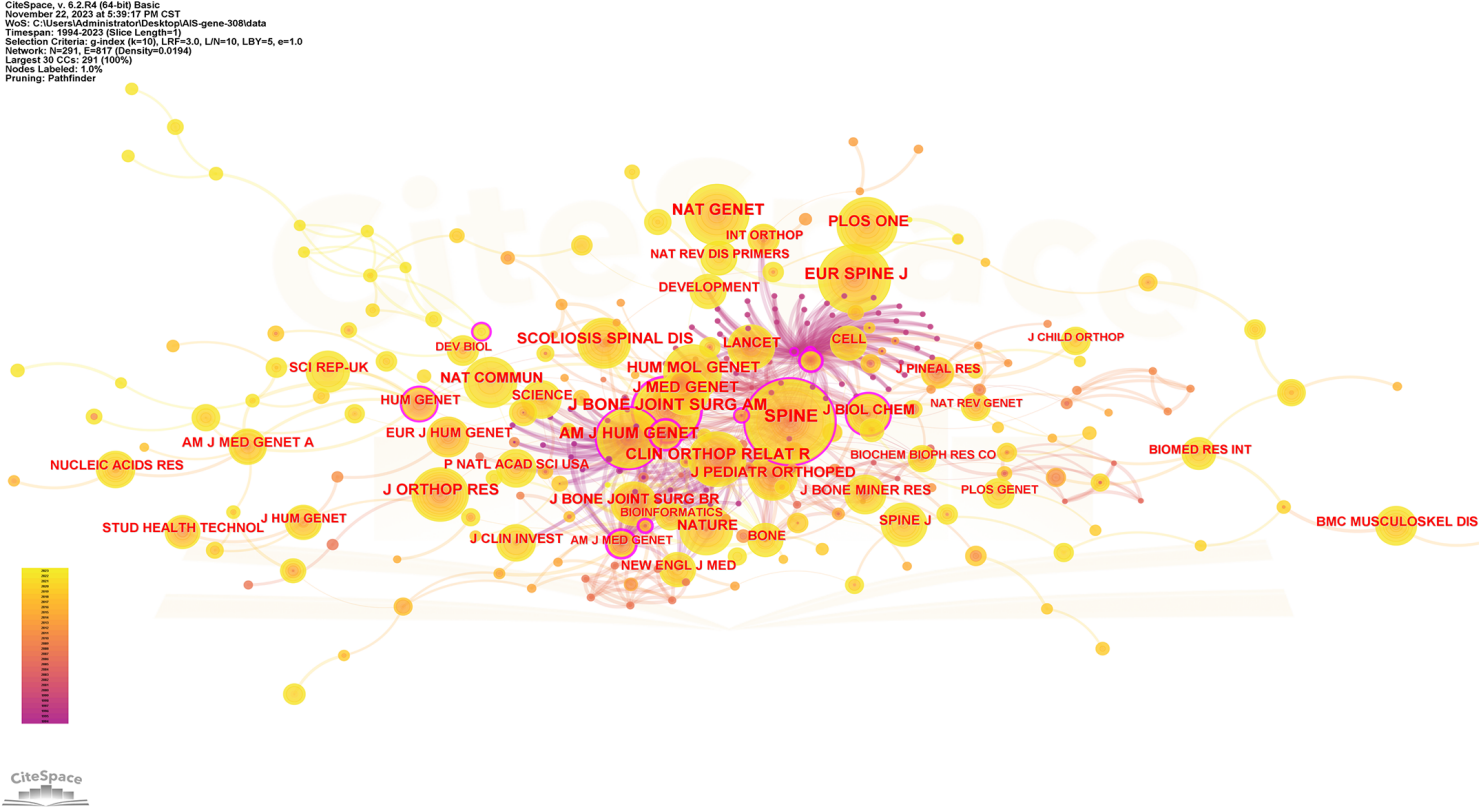
Collaboration network of cited journals. Nodes represent journals, lines represent co-citation relationships. Node colors indicate different years. Node size reflects the number of co-citations.

Table 3 shows the ranking of journals that published articles on AIS genetic factors. Most papers were published in *Spine* (62; 20.1%), followed by *European Spine Journal* (17; 5.5%) and *BMC Musculoskeletal Disorders* (11; 3.6%), *Scientific Reports* (11; 3.6%) and *Human Molecular Genetics* (8; 2.6%). They accounted for 35.4% of all publications. Figure 5 shows the cooperation among these journals.

**Table 3:** Top 10 productive journals in the genetic factors of AIS.

Rank	Journal	Number	Percentage
1	Spine	62	20.1
2	European Spine Journal	17	5.5
3	BMC Musculoskeletal Disorders	11	3.6
4	Scientific Reports	11	3.6
5	Human Molecular Genetics	8	2.6
6	Journal of Orthopaedic Research	8	2.6
7	PLOS ONE	7	2.3
8	Spine Journal	7	2.3
9	American Journal of Medical Genetics Part A	5	1.6
10	Biomed Research International	5	1.6

### Author analysis

**Figure 6:**
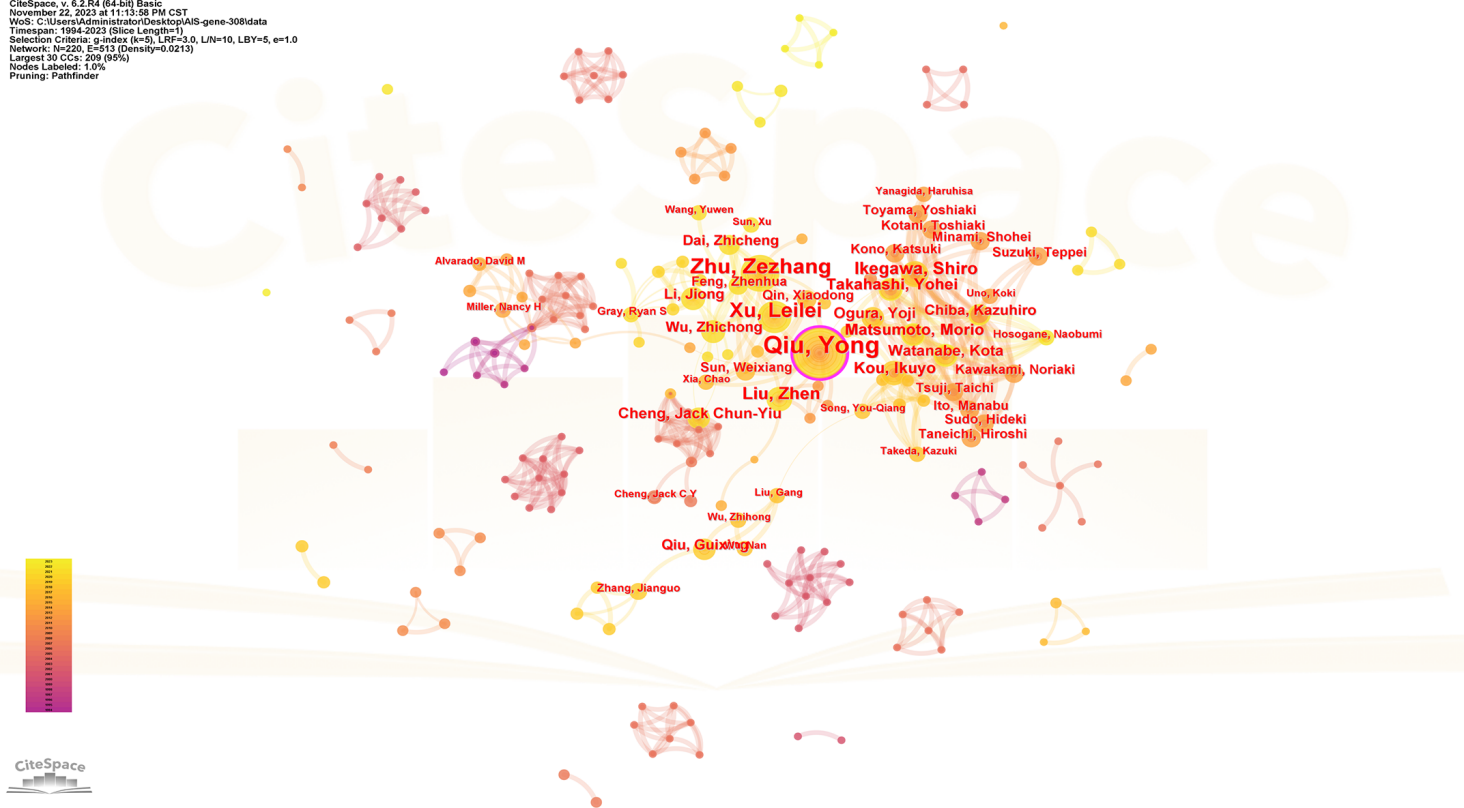
Collaboration network of productive authors. Nodes represent authors, lines represent collaborations. Node colors indicate different years. Node size reflects publication count.

**Figure 7:**
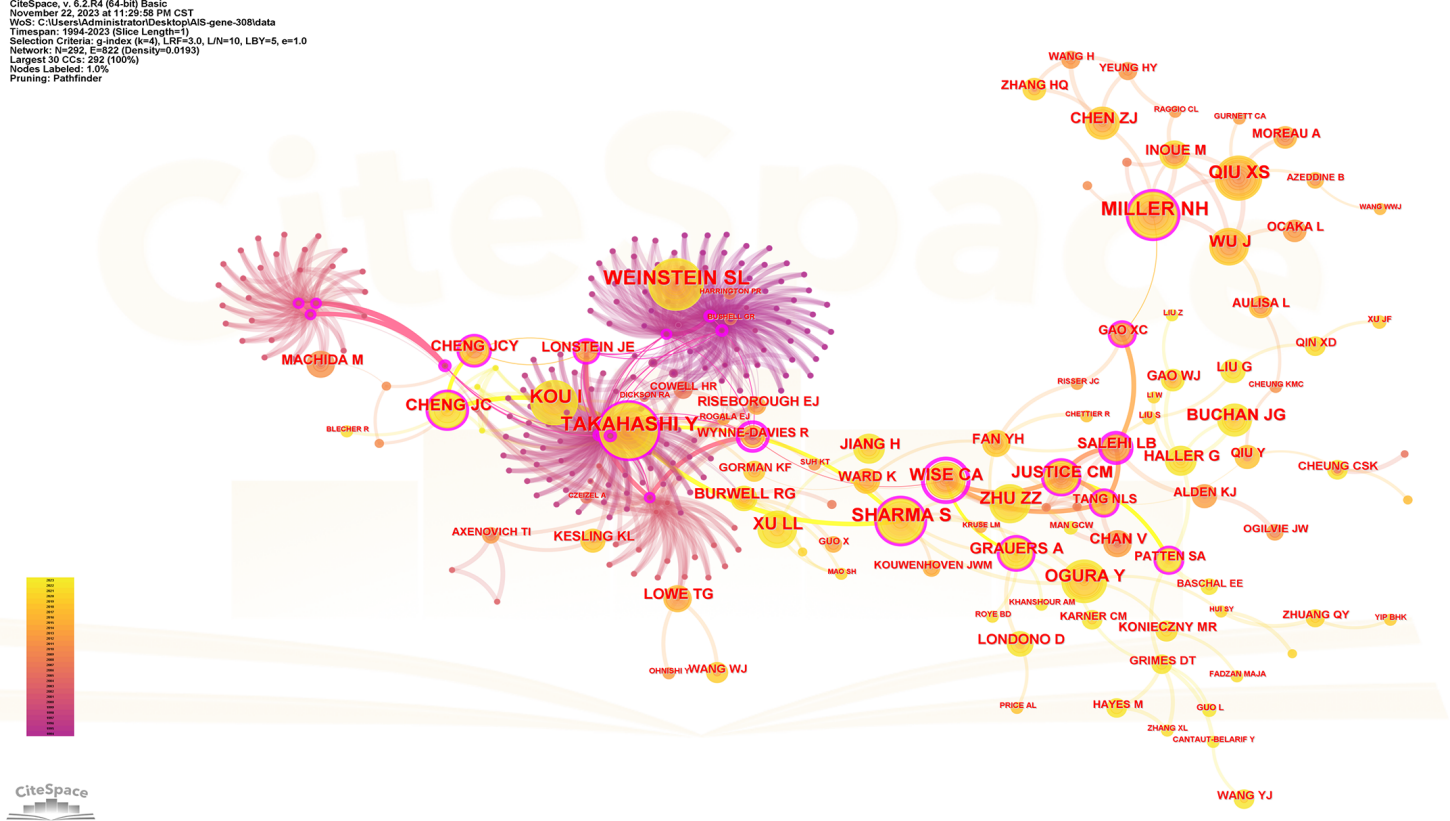
Collaboration network of cited authors. Nodes represent co-cited authors. Lines represent co-citation relationships. Node colors indicate different years. Node size reflects the number of co-citations.

One thousand five hundred five scholars participated in the publication of 308 articles. Table 4 shows the 10 most prolific scholars. Figure 6 presents the collaboration network of the scholars. Y. Qiu and Z. Z. Zhu contributed to the publication of 59 and 36 papers respectively. The top 3 most cited scholars are Y. Takahashi (113), S. L. Weinstein (111) and N. H. Miller (105). When 2 articles refer to the same article, a common reference link is created. Figure 7 shows the influential authors associated with the genetic factors of AIS. Authors or co-authors of papers working in identical nations appear in the bibliographic link.

**Table 4:** The most productive authors in the genetic factors of AIS.

Rank	Author	Number	Percentage	Affiliation
1	Y. Qiu	59	19.2	The Affiliated Drum Tower Hospital of Nanjing University Medical School, Nanjing, China.
2	Z. Z. Zhu	36	11.7	The Affiliated Drum Tower Hospital of Nanjing University Medical School, Nanjing, China.
3	L. L. Xu	32	10.4	The Affiliated Drum Tower Hospital of Nanjing University Medical School, Nanjing, China.
4	J. Cheng	27	8.8	Joint Scoliosis Research Center of The University of Hong Kong, China.
5	S. Ikegawa	21	6.8	Laboratory of Bone and Joint Diseases, Center for Genomic Medicine, RIKEN, Tokyo, Japan.
6	Z. Liu	20	6.5	The Affiliated Drum Tower Hospital of Nanjing University Medical School, Nanjing, China.
7	K. Ikuyo	17	5.5	Laboratory of Bone and Joint Diseases, Center for Integrative Medical Sciences, RIKEN, Tokyo, Japan.
8	G. Christina	15	4.9	Department of Orthopaedic Surgery, Washington University School of Medicine, St. Louis, MO, USA
9	N. L. Tang	14	4.5	Departments of Chemical Pathology, University of Hong Kong, China.
10	Y. Ogura	14	4.5	Department of Orthopaedic Surgery, Keio University School of Medicine, Tokyo, Japan.

### Reference analysis

**Figure 8:**
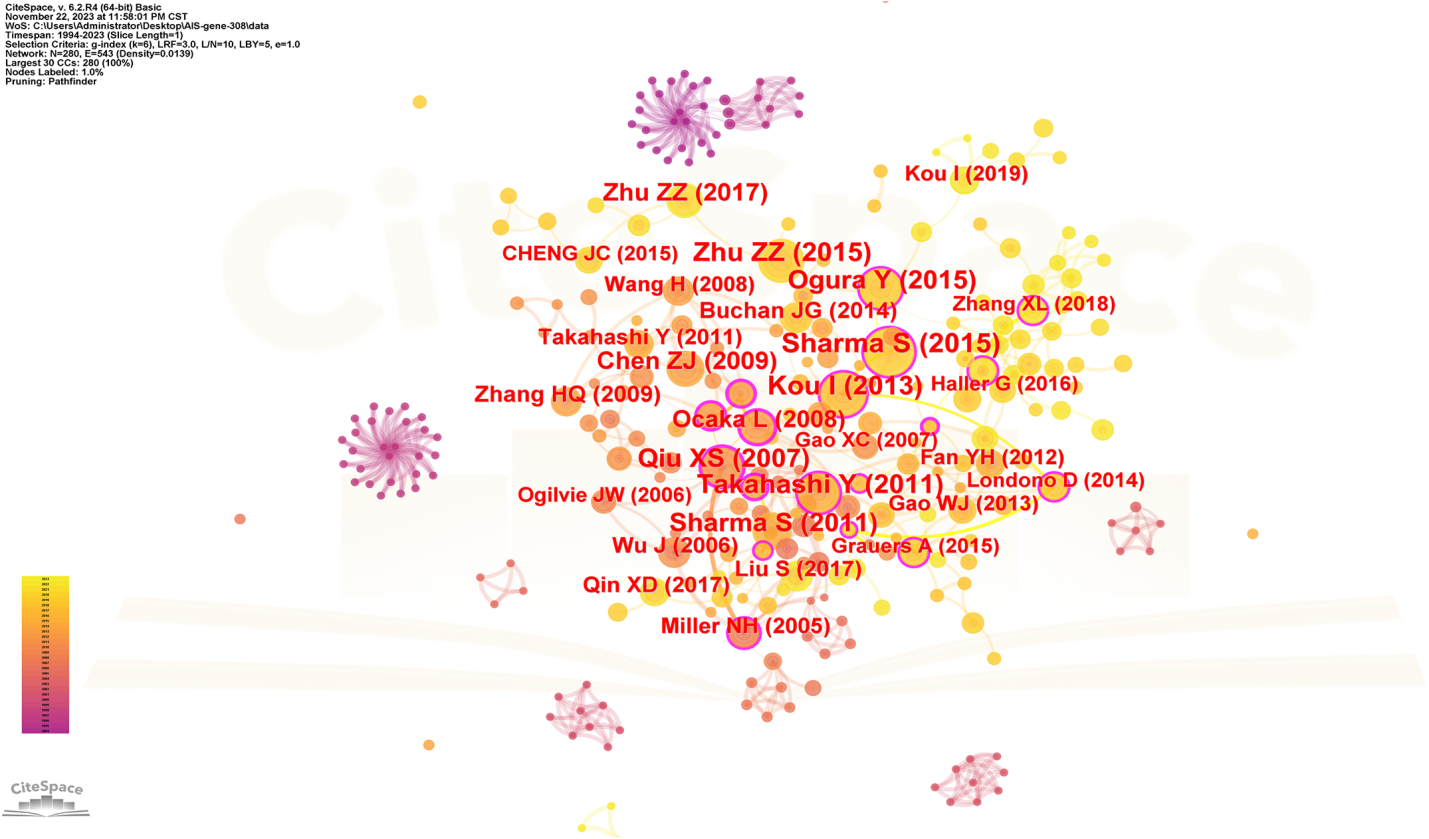
The network of cited references. Nodes represent references, lines represent co-citation relationships. Node colors indicate different years. Node size reflects the number of co-citations.

The spacing of references in co-citation analysis reveals the co-citation relationship. Table 5 enumerates the top 10 cited articles regarding genetic factors in AIS. Among the 10 articles, 4 originate from China, 3 from Japan, 2 from the United States, and 1 from the United Kingdom. Figure 8 displays the network of cited references, depicting the co-citation relationships among them.

**Table 5:** Top 10 cited articles in the genetic factors of AIS.

Rank	Title	Authors	Corresponding author's country	Journal	Year	Citation
1	A *PAX1* enhancer locus is associated with susceptibility to idiopathic scoliosis in females	S. Sharma et al	United States	Nature Communication	2015	36
2	Genetic variants in *GPR126* are associated with adolescent idiopathic scoliosis	K. Ikuyo et al	Japan	Nature Genetics	2013	34
3	Genome-wide association study identifies new susceptibility loci for adolescent idiopathic scoliosis in Chinese girls	Z. Z. Zhu et al	China	Nature Communication	2015	33
4	A genome-wide association study identifies common variants near *LBX1* associated with adolescent idiopathic scoliosis	Y. Takahashi et al	Japan	Nature Genetics	2011	30
5	Genome-wide association studies of adolescent idiopathic scoliosis suggest candidate susceptibility gene	S. Sharma et al	United States	Human Molecular Genetics	2011	30
6	Melatonin Receptor 1B (*MTNR1B*) Gene Polymorphism Is Associated With the Occurrence of Adolescent Idiopathic Scoliosis	X. S. Qiu et al	China	Spine	2007	29
7	A Functional SNP in *BNC2* Is Associated with Adolescent Idiopathic Scoliosis	Y. Ogura et al	Japan	The American Journal of Human Genetics	2015	28
8	Promoter polymorphism of matrilin-1 gene predisposes to adolescent idiopathic scoliosis in a Chinese population	Z. J. Chen et al	China	European Journal of Human Genetics	2009	24
9	Genome-wide association study identifies novel susceptible loci and highlights *Wnt/β-catenin* pathway in the development of adolescent idiopathic scoliosis	Z. Z. Zhu et al	China	Human Molecular Genetics	2017	24
10	Assignment of two loci for autosomal dominant adolescent idiopathic scoliosis to chromosomes 9q31.2-q34.2 and 17q25.3-qtel	L. Ocaka et al	United Kingdom	Journal of Medical Genetics	2008	21

### Keyword analysis and research hotspots

**Figure 9:**
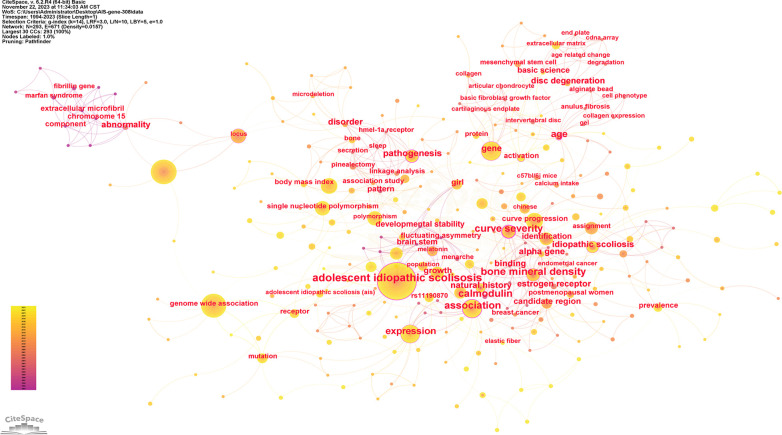
The network of keywords. Nodes represent keywords, lines represent co-occurrence relationships. Colors in the nodes indicate different years. Node size reflects its frequency.

**Figure 10:**
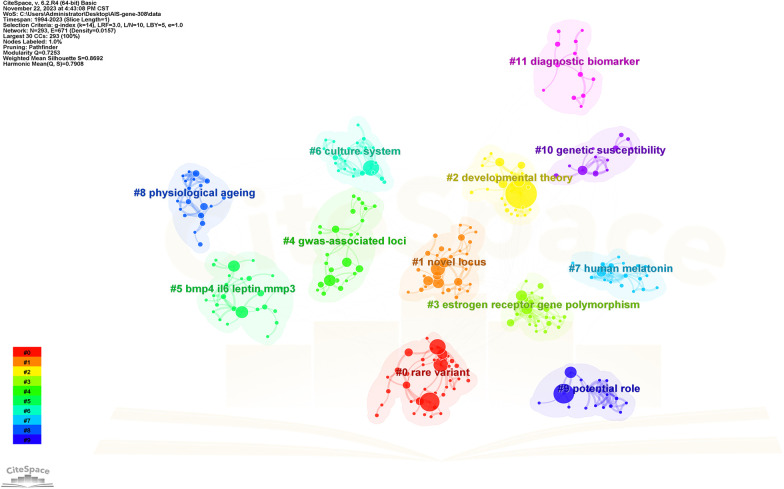
The cluster view map. Each grid contains a cluster of locations. The lower the number, the higher the number of keywords included in the clusters.

Keywords serve as indicators of the study's subject matter. Summarizing frequently occurring and highly prominent keywords proves useful for describing research focal points and trends. The larger the nodes within the keyword co-occurrence map, the more substantial the keyword weight. Enhanced connections are portrayed by reduced distances between nodes. Bolder lines signify an amplified frequency of 2 phrases being referenced simultaneously. Figure 9 presents the network of keywords. Keyword clustering involves grouping words and phrases that exhibit clear domain characteristics. It utilizes feature extraction algorithms to classify text and perform domain-based clustering of words. By managing word frequency, it identifies both general and specific domain-related terms. Figure 10 shows the 12 clusters in this study: “rare variant” #0, “novel locus” #1, “developmental theory” #2, “estrogen receptor gene polymorphism” #3, “GWAS-associated loci” #4, “BMP4 IL6 leptin MMP3” #5, “culture system” #6, “human melatonin” #7, “physiological aging” #8, “potential role” #9, “genetic susceptibility” #10 and “diagnostic biomarker” #11.

**Figure 11:**
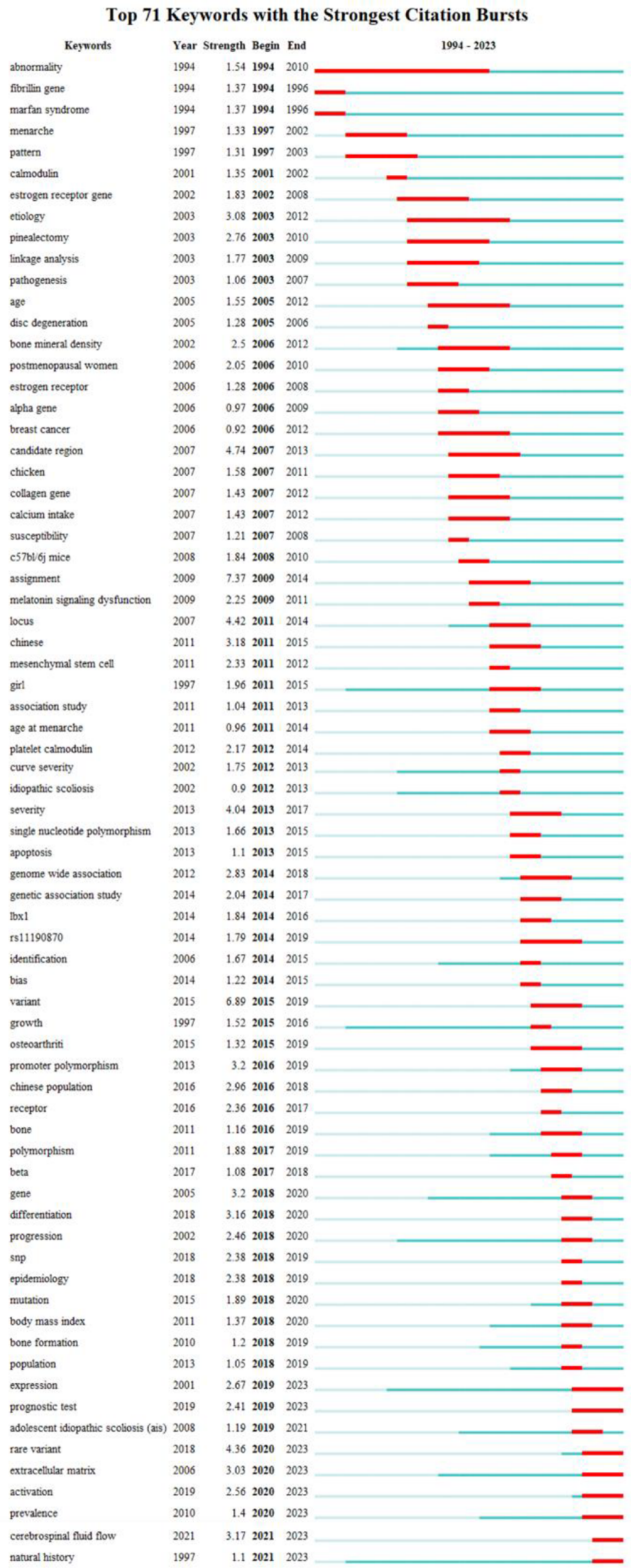
Top 71 keywords with the strongest citation bursts. The red bar is the burst phase.

Figure 11 presents the top 71 cited keywords. The red and blue bars indicate common and uncommon keywords correspondingly. The most popular keywords in AIS genetic factors were “fibrillin gene”, “menarche”, “calmodulin”, “estrogen receptor gene”, “linkage analysis”, “disc degeneration”, “bone mineral density”, “melatonin signaling dysfunction”, “collagen gene”, “mesenchymal stem cell”, “*LBX1*”, “promoter polymorphism”, “Bone formation”, “cerebrospinal fluid flow” and “extracellular matrix”.

## Discussion

The research showcases the results of a bibliometric analysis of 308 articles on AIS genetic factors published from January, 1, 1992, to February, 28, 2023 utilizing the WoS database and CiteSpace software. The evolution from 1992 to 2023 is described by 2 phases: 1992–2005, a period of slow growth, and 2006–2023, a phase of swift progress. The number of papers published in 2019 is the largest, but this does not mean that the field has reached its peak. Scholars persist in their field with ongoing research, promising more influential publications ahead.

Analysis of the distribution of countries, institutions and scholars shows international collaboration in this field. According to the article count and the H-index, China stood out as the foremost nation in terms of productivity and impact in AIS genetic factor research. Leveraging its sizable population and proficiency in sample collection from AIS patients, China has witnessed accelerated development in studying AIS genetic factors, propelled by economic, technological, and intellectual advancements. The advancement of the economy has stimulated investment in the healthcare sector, leading to a surge in research productivity (6, 9). In addition, young Chinese spine surgeons excel in basic research and article publication. The USA, a powerhouse of economy, science, and technology, assumes a vital role in studying the AIS genetic factors. When analyzing the dispersion of academic papers by nation, organization, and author, Japan was also a noteworthy participant in research efforts.

Qiu Yong and Zhu Zezhang, affiliated with Drum Tower Hospital, Nanjing University, China, have published extensively on this topic. They have maintained a close collaboration. The quantity of co-authors serves as a significant metric for the status of the research. The visual map of this study indicates inadequate connections among countries, institutions, and authors. Enhancing academic collaboration is imperative.

Journal analysis assists researchers in selecting appropriate publications. Spine and European Spine Journal are the mainstream journals on the topic. These journals might exhibit greater openness to researching genetic factors in AIS, and manuscripts featured in these journals have higher chances of gaining attention and citations.

Keywords reflect the focus and trajectory of the research. Keyword evolution analysis provides a crucial basis for exploring the research of AIS genetic factors. After removing keywords related to the topic, the most prominent burst keywords of AIS genetic factors are “fibrillin gene”, “menarche”, “calmodulin”, “estrogen receptor gene”, “linkage analysis”, “disc degeneration”, “bone mineral density”, “melatonin signaling dysfunction”, “collagen gene”, “mesenchymal stem cell”, “*LBX1*”, “promoter polymorphism”, “Bone formation”, “cerebrospinal fluid flow” and “extracellular matrix”. The results of the burst keywords and clustering revealed that the prominent theme in this field over the last 30 years has been the expression and verification of genes related to the spine and its accessory structures.

Most of the 10 most cited papers investigated the genetic factors of AIS through genome-wide association studies. In 2007, Xu et al. found that the *MTNR1B* gene is lowly expressed in osteoblasts from AIS patients but is not involved in disease progression (10). Subsequently, AIS-related susceptibility genes continue to emerge, including *MATN1* (11), *LBX1* (12), *PAX1* (13), *BNC2* (14) and *GPR126* (15, 16). Except for the *BNC2* gene, all genes were lowly expressed in the tissue cells of AIS patients. AIS-related signaling pathways have also shown remarkable results. Sharma et al. found a possible association between the clinical phenotype of horizontal gaze palsy with progressive scoliosis in patients with compound heterozygous mutations in *ROBO3* and the involvement of the *ROBO3* gene in the regulation of the axon guidance pathway (17). Zhu et al. found that the proportion of type I fibers was higher on the convex side than on the concave side in patients with AIS, which may be related to the asymmetric expression of the Wnt/*β*-catenin pathway in the paraspinal muscles on both sides of the spine (18). These studies suggest that AIS may be the result of the combined effects of multiple gene expression and different signaling pathways.

Combining keyword burst analysis and keyword clustering analysis, we summarized the new perspectives of AIS genetic factors research papers published in the last 5 years as: urotensin and its related receptors, cilia, chondrocytes and osteogenesis, muscle tissue, neural crest cells, and connective tissue.

### Urotensin and its related receptors

Zebrafish, genetically similar to humans, are ideal subjects for studying scoliosis due to their inherent susceptibility to the condition (19, 20). The circulation of cerebrospinal fluid (CSF) is associated with spinal development and can result in zebrafish developing spinal deformities (21–23). When CSF flows through neurons in contact with CSF(CSF-cNs) in the central canal, these neurons produce the urotensin-related peptides Urp1 and Urp2 (24–26). These peptides can bind to Uts2r receptors on the membrane of dorsal slow-twitch myocytes in zebrafish embryos, causing the myocytes to contract dorsally and straighten the body axis. Reducing these peptides during zebrafish growth causes spinal deformities similar to human spinal dysplasia (27, 28). Bearce et al. found that zebrafish lacking Uts2r3 have scoliosis in adulthood; and that Uts2r3, as a receptor of the urotensin family, may regulate spinal morphology (21). Research on Chinese Han patients with AIS has additionally verified a substantial correlation between *UTS2R* gene mutation and AIS (29). It can be concluded that mutations in the genes that control the production of urotensin and its receptor may lead to the development of AIS.

### Cilia

Cilia are hairy organelles positioned outside the cell membrane (30). Active cilia produce cellular motility, drive fluid flow or generate signal gradients (22, 31). Cilia assembly and CSF flow are closely associated with AIS (22). Elizabeth et al. suggest that motile cilia in the zebrafish vertebral canal promote the synthesis of Reissner's fibers, which transport epinephrine to the central canal. Epinephrine acts on CSF-cNs to induce Urp peptides secretion. These urotensins signal the zebrafish dorsal slow-twitch muscles to elicit contractions that resolve the ventral curve and promote spinal straightening (28). The mutation rate of the *POC5* gene in French-Canadian and British AIS patients was significantly higher than in normal controls (32). Its aberrant expression affects Urp peptides secretion, which limits the contraction of slow-twitch muscles and imbalances muscle strength on both sides of the spine, causing scoliosis (33). Similarly, variants in the *TTLL11* gene were present in the majority of UK families with concomitant scoliosis; a zebrafish model with the gene knocked out replicated this condition (34). Abnormal ciliary movement may also lead to scoliosis. Zebrafish cilia contribute to Reissner fiber aggregation and straightening of the body axis; and knockout of the ciliary polarity and movement-related gene *cfap298* (35) and ciliary dynamic protein axonal heavy chain 10 genes may affect Reissner fiber aggregation and lead to zebrafish spinal deformity (21, 36). Variants in genes *dnaaf1* and *zmynd10* related to cilia structure and function were also found in AIS patients in southern China by whole exome sequencing (37). Knockout of these genes in viable adult zebrafish recapitulated scoliosis (37). It can be concluded that mutations in cilia-related genes cause AIS by impeding CSF flow, Reissner fiber synthesis, and urotensin release allowing an imbalance of paraspinal musculature.

### Chondrocytes and osteogenic process

Meta-analyses have summarized and confirmed that AIS-related gene mutations affect chondrocyte development and osteogenesis (such as *CDH13*, *ABO* and *COMP*) (38, 39). Among them, *COMP* gene expression is down-regulated in osteoblasts of AIS patients, which synthesize abnormal proteins with cytotoxicity, triggering chondrocyte underdevelopment or even death, resulting in impaired bone growth (39). A number of studies on signaling pathways involved in the process. The *ERK1/2* signaling pathway activates the *AKAP2* gene, promoting the proliferation and specialization of chondrocytes in the human growth plate, while *AKAP2* gene expression was reduced in AIS patients (40). Upregulation of the *RHOA* gene in AIS patients inhibits the differentiation of MSCs to cartilage through the RHOA/ROCK signaling pathway and hinders bone growth (41). *SPRY4* gene is the key to promoting osteogenic differentiation and melatonin response in mesenchymal stem cells. Its overexpression, along with melatonin, enhances osteogenesis, whereas knockout of *SPRY4* hampers osteogenesis in AIS patients (42, 43). Low expression of *ADGRG7*, *GREM1* and *GPR126* genes affects osteogenesis in AIS patients (44–47). *CHD7* and *BOC* gene expression even positively correlated with bone mineral content in AIS patients (48, 49).

### Muscle tissue

AIS is often accompanied by differential expression of genes in paraspinal muscle cells. The abnormal expression of the *LBX1* gene has been replicated in a large sample of multi-ethnic AIS patients (50, 51) and successfully verified in mouse experiments (52). Low expression of the *LBX1* gene may reduce energy supply to skeletal muscle cells and accelerate disease progression by affecting galactose metabolism and glycolytic pathways (53). Genes also regulate the development of paraspinal muscles. High expression of the *TENT5A* gene facilitates myofiber maturation by promoting the proliferative migration of myofibroblasts and maintaining the stability of myogenin; whereas, the *TENT5A* gene is lowly expressed in the paraspinal muscles of patients with AIS (54). In addition to the genes mentioned above, numerous other genes are lowly expressed in the paraspinal muscles of AIS patients, and their functions have yet to be investigated, including *PIEZO2 (*55), *CDH13* (56), *ABO* (57), *SLC39A8* (58), *ROBO3* (59), *IRX1* (60), *H19* (61) and *SOCS3* (62).

### Neural crest cell

AIS patients displayed chondroblasts in the curved convex growth plate, while the concave side had neuroblasts and glial blasts (63). Bilateral growth imbalance results in scoliosis. Neural crest cells migrate along a specific pathway during embryonic development to form neuroblasts and glial blasts (63, 64). During migration, neural crest cells experience epithelial-mesenchymal transition (65), facilitating their migration into the mesenchymal extracellular matrix (66). *PAX3* gene is linked to the formation of mesenchymal extracellular matrix, including the expression of two multifunctional proteoglycan subtypes (V1 and V0) (67). Multifunctional proteoglycan can guide the migration of neural crest cells (68, 69). Cartilage differentiation of mesenchymal cells may influence the migration of neural crest cells (70). Therefore, the *PAX3* gene may cause non-synchronous migration of neural crest cells and different phenotypes of cells on both sides of the spine, resulting in idiopathic scoliosis.

### Connective tissue

Connective tissue plays a role in maintaining normal spine morphology, including intervertebral discs, ligaments, and tendons (71, 72). The *ADGRG6* gene regulates the biomechanical structure of intervertebral discs and dense connective tissues to maintain normal spinal morphology through the cAMP/CREB signaling pathway; whereas AIS patients have defective expression of the *ADGRG6* gene (73). The expression of the *PAX-1* gene in the disc is involved in the formation of the spinal structure (74). In addition, upregulation of *ERC2* and *MAFB* gene expression in AIS patients may promote hypertrophy of the ligamentum flavum to adapt to mechanical stresses generated by scoliosis via the TGF-β pathway (75). The *FBN1* gene is crucial for connective tissue function (76), and its low expression reduces the synthesis of extracellular matrix proteins, which is detrimental to maintaining the stability of the biomechanical structure of connective tissues such as ligaments and intervertebral discs, leading to the progression of AIS (77). ADAMTSL2 and LTBPs can bind to FBN1 *in vitro* and upregulate the TGF-β signaling pathway in fibroblasts (78–81). Ryzhkov et al. found that the TGF-β signaling pathway, which is involved in the formation and degradation of extracellular matrix proteins, is more highly expressed on the concave side of the curve than on the convex side in patients with AIS, and may be involved in the process of disc tissue degeneration (82). Therefore, The interaction between ADAMTSL2 and LTBP4 is involved in the development of AIS through the TGF-β pathway (83). The above results show that connective tissue-related genes are involved in the onset and development of AIS.

Although research on genetic factors associated with AIS is recent and extensive, most studies are not in-depth and have some limitations.

1. Most studies have small sample sizes and selection bias, and the samples can not represent the patient group (33, 84).
2. Many studies only examine whether there is a correlation, but do not explain the causality and mechanism of action (14, 16).
3. Some studies are unable to obtain muscle, bone, and other tissues that may be directly related to AIS (85, 86).
4. Integrating the existing research results and establishing the mathematical model of AIS is beneficial for the diagnosis and prevention of the disease (87).

In the process of studying the etiology of AIS, researchers have gradually recognized that AIS is a disease caused by the combined effects of multiple factors. Over the past few decades, the study of AIS etiology has shifted from a macroscopic to a microscopic level, particularly with the rise of GWAS, which allows for comprehensive screening of genes associated with the occurrence and development of AIS. As a result, various tissues related to AIS have been included in the research. While these studies have provided abundant results, they have been scattered, making it difficult to identify the primary effect genes. Additionally, there is a lack of AIS samples from diverse ethnic backgrounds, preventing integration into a unified system.

Artificial intelligence (AI) is well known for its ability to analyze and process large amounts of data, and discover hidden relationships between variables. By consolidating clinical and GWAS data from previous AIS patients and constructing a database that is continuously updated, we can combine the vast clinical information and GWAS data of AIS patients with AI. This integration allows for the training of a dynamic AIS simulation system, which can uncover associations between potential genetic variations and specific clinical phenotypes, predict AIS risk and progression, simulate gene-targeted therapies for AIS, and even customize personalized disease risk assessment models based on individual AIS patients' genomic information and relevant clinical parameters. This approach provides targeted medical advice and treatment plans, contributing to the development of precision medicine and improving treatment outcomes and health management for each patient.

### Advantages and constraints of this research

This is an inaugural bibliometric analysis of the genetic factors of AIS. This study discusses the current situation, progress and trend of the role of genetic factors in AIS, so that scholars can focus on the latest and most important hotspots. This study has several limitations. First, there is a temporal delay as this study did not incorporate recent publications. Second, it is not comprehensive. We only looked at articles from the WoS core collection. Finally, subjective bias exists in the interpretation of the results.

## Conclusion

Research into the genetic factors of AIS indicates a worldwide pattern over time. Beginning in 2006, there has been a significant surge in the quantity of published research. China and the Chinese Academy of Medical Sciences emerge as the most productive countries and institutions in this regard. A large number of articles have been published in *Spine* and *European Spine Journal*. Y. Qiu and Z. Z. Zhu are both prolific authors. The strongest burst keywords among the genetic factors of AIS were “fibrillin gene”, “menarche”, “calmodulin”, “estrogen receptor gene”, “linkage analysis”, “disc degeneration”, “bone mineral density”, “melatonin signaling dysfunction”, “collagen gene”, “mesenchymal stem cell”, “*LBX1*”, “promoter polymorphism”, “Bone formation”, “cerebrospinal fluid flow” and “extracellular matrix”. In addition, scholars would find valuable insights from the prominent clusters, highly cited publications and references.
